# Peculiarities and pitfalls of quantifying mitochondrial energy metabolism in the skin

**DOI:** 10.1111/exd.12895

**Published:** 2016-01-12

**Authors:** René G. Feichtinger, Barbara Kofler

**Affiliations:** ^1^ Laura Bassi Centre of Expertise – THERAPEPResearch Program for Receptor Biochemistry and Tumor MetabolismDepartment of PediatricsParacelsus Medical UniversitySalzburgAustria

**Keywords:** keratinocyte, mitochondria, oxidative phosphorylation

In the last few years, mitochondria have exited from the shadows to take centre stage in the aetiology of several skin disorders. Thus, the role of mitochondrial energy metabolism in healthy and diseased skin has become a hot topic in dermatology 1. For example, classic skin disorders affecting intermediate filaments such as epidermolysis bullosa can now be linked to mitochondrial pathophysiology 2. However, the complex morphology of the epidermis presents difficulties in analyses of skin mitochondrial function, especially of energy metabolism in keratinocytes. Forni et al. 2015 reported on the bioenergetic profiling of mouse skin and measured respiration of isolated mitochondria from whole skin homogenates 3. However, their method actually generates only an average readout from different cell types, including keratinocytes, fibroblasts, immune cells, nerve cells, endothelial cells of vessels to name just some. Moreover, this limitation highlights that the frequent demand by reviewers to provide functional data is sometimes inappropriate, because the enzymatic/functional measurements of heterogeneous tissues do not reflect the situation at the cellular level *in vivo*.

These pitfalls can be at least partly circumvented by immunohistochemical staining of tissue samples. Numerous reports demonstrate there is excellent correlation between the amount of expression at the protein level and the activity of oxidative phosphorylation (OXPHOS) enzymes 4, 5. Indeed, the activity of OXPHOS complexes is regulated mainly by the amount of protein expressed and less so by protein modifications like phosphorylation. However, the selection of appropriate antibodies is of great importance. Because OXPHOS complexes are assembled out of many subunits, it is important when choosing antibodies for immunohistochemistry to select ones directed against subunits incorporated at a very late stage of assembly. Furthermore, the targeted subunit should be one that is degraded when it is not assembled into the OXPHOS complex. In addition to detecting the subunit targeted by the antibody, other subunits that are incorporated earlier than the targeted subunit can be detected indirectly by this approach; this is because absence of several subunits (e.g. as a consequence of mutation) usually leads to degradation of the entire OXPHOS complex.

Forni et al. 2015 also measured oxygen consumption in isolated keratinocytes and fibroblasts 3. However, another important factor they did not consider is that even isolated pure keratinocytes of the skin still do not resemble the *in vivo* situation, because keratinocytes, especially in human skin, are physiologically heterogeneous. Usually, basal keratinocytes, which can be regarded as young cells, have a higher content of mitochondria and OXPHOS enzymes than do peripheral keratinocytes, which could be a consequence of the fact that mitochondria typically degrade along the basal/apical axis (Fig. 1) 6.

**Figure 1:**
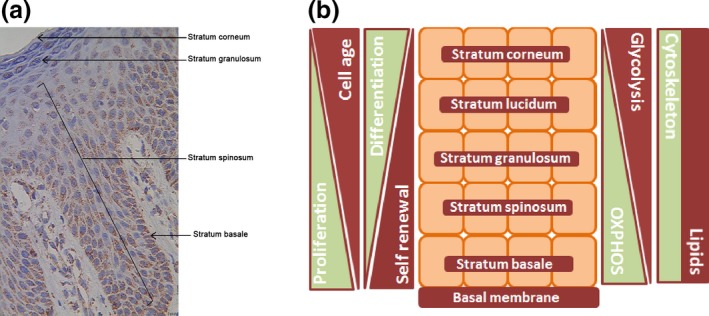
Distribution of mitochondria in epidermis and schematic illustration of metabolic differences in layers of the epidermis. (a) Expression of the mitochondrial membrane protein porin, a marker for mitochondrial mass, in normal human epidermis. (b) Schematic drawing depicting differences in skin physiology associated with different layers of the epidermis. According to the literature, mitochondrial fragmentation occurs in the epidermis 6. The most degraded mitochondria are found in the outer layers. Therefore, oxidative phosphorylation (OXPHOS) should decrease and glycolysis increase from the stratum basale to the stratum corneum. Self‐renewal capacity and proliferative potential both decrease from the basal to the apical layer, whereas both differentiation and age of the keratinocytes increase. Cytoskeletal proteins and lipid composition likewise differ in the layers (s14, s15). Porin staining was performed as previously described (s16).

Parameters like skin thickness, age and localization might also influence the functional outcome significantly and may partly explain the regional disparities within the epidermis. Moreover, extrapolation of data from mice to humans is fraught with problems, as murine skin contains a substantially higher number of hair follicles, is thinner and is composed of fewer layers.

Determination of energy metabolism in primary cultures of skin fibroblasts is performed routinely in the diagnostic workup of patients with mitochondrial disorders 7. However, whether keratinocytes are an alternative to fibroblasts for diagnostic purposes is questionable. Isolation of keratinocytes and their cultivation are difficult, and because in culture mainly stem‐cell‐type keratinocytes will be isolated, measurement of energy metabolism in such cells offers no advantage over the gold standard fibroblasts, and the results obtained could be misleading.

More work is required on many more skin samples to evaluate whether there are anatomically regional, sex or age differences regarding mitochondrial metabolism in keratinocytes. In addition, a selection bias might be present during keratinocyte isolation. Depending on the preparation method and localization or thickness of the epidermis, keratinocytes with either high or low mitochondrial energy metabolism might be preferentially isolated. Moreover, keratinocytes with a high aerobic mitochondrial energy metabolism could have a selective growth advantage and possibly a higher capacity for self‐renewal and might therefore overgrow cells from other layers during preparation of primary cultures for subsequent analysis. Therefore, immunohistochemical analysis is the method of choice to overcome the challenge of tissue/cell‐type variability.

Very recently, several interesting issues regarding skin physiology were addressed, showing that mitochondria play a role in hair follicle development 8, proliferation of epidermal progenitor cells 9, and melanocyte function and pigmentation (s10). Furthermore, modulation of mitochondrial function in the skin and other tissues by endogenous regulatory peptides and hormones is considered to be important in tissue homeostasis and may even be involved in counteracting the age‐related decline of mitochondrial function (s11–13).

It is now clear that skin physiology and mitochondrial energy metabolism are more intimately linked than was previously thought, a connection that calls for a thorough analysis at the cellular level. As this link was recognized just recently, further exciting discoveries can be expected within the next few years concerning the affair between the skin and mitochondria.

## Author contribution

RGF performed research, and RGF and BK designed the study and wrote the paper.

## Conflict or interests

The authors have declared no conflicting interests.

## Supporting information


**Data S1.** References.
