# Supplementary material for: Peculiarities and pitfalls of quantifying mitochondrial energy metabolism in the skin
Source: Exp Dermatol. 2016 Jan 12;25(2):101–2. doi: 10.1111/exd.12895 (PMC4738473; doi:10.1111/exd.12895)
Supplement: Supplementary file 1 — Data S1. References. [file EXD-25-101-s001.docx]

**Supplementary references**

**s10** Ni-Komatsu L, Orlow S J. J Invest Dermatol 2007: **127**: 1585-1592.

**s11** Paus R, Langan E A, Vidali S*, et al.* Trends Mol Med 2014: **20**: 559-570.

**s12** Poeggeler B, Schulz C, Pappolla M A*, et al.* Exp Dermatol 2010: **19**: 12-18.

**s13** Vidali S, Knuever J, Lerchner J*, et al.* J Invest Dermatol 2014: **134**: 33-42.

**s14** Natarajan V T, Ganju P, Ramkumar A*, et al.* Nat Chem Biol 2014: **10**: 542-551.

**s15** Simpson C L, Patel D M, Green K J. Nat Rev Mol Cell Biol 2011: **12**: 565-580.

**s16** Feichtinger R G, Weis S, Mayr J A*, et al.* Glia 2014: **62**: 514-525.
